# Neurotrophic Factors in Parkinson’s Disease: Clinical Trials, Open Challenges and Nanoparticle-Mediated Delivery to the Brain

**DOI:** 10.3389/fncel.2021.682597

**Published:** 2021-06-02

**Authors:** Olesja Bondarenko, Mart Saarma

**Affiliations:** ^1^Institute of Biotechnology, HiLIFE, University of Helsinki, Helsinki, Finland; ^2^Laboratory of Environmental Toxicology, National Institute of Chemical Physics and Biophysics, Tallinn, Estonia

**Keywords:** Parkinson’s disease, brain-derived neurotrophic factor, cerebral dopamine neurotrophic factor, dopamine neurons, endoplasmic reticulum stress, glial cell line-derived neurotrophic factor, nanomedicine, neuroinflammation

## Abstract

Neurotrophic factors (NTFs) are small secreted proteins that support the development, maturation and survival of neurons. NTFs injected into the brain rescue and regenerate certain neuronal populations lost in neurodegenerative diseases, demonstrating the potential of NTFs to cure the diseases rather than simply alleviating the symptoms. NTFs (as the vast majority of molecules) do not pass through the blood–brain barrier (BBB) and therefore, are delivered directly into the brain of patients using costly and risky intracranial surgery. The delivery efficacy and poor diffusion of some NTFs inside the brain are considered the major problems behind their modest effects in clinical trials. Thus, there is a great need for NTFs to be delivered systemically thereby avoiding intracranial surgery. Nanoparticles (NPs), particles with the size dimensions of 1-100 nm, can be used to stabilize NTFs and facilitate their transport through the BBB. Several studies have shown that NTFs can be loaded into or attached onto NPs, administered systemically and transported to the brain. To improve the NP-mediated NTF delivery through the BBB, the surface of NPs can be functionalized with specific ligands such as transferrin, insulin, lactoferrin, apolipoproteins, antibodies or short peptides that will be recognized and internalized by the respective receptors on brain endothelial cells. In this review, we elaborate on the most suitable NTF delivery methods and envision “ideal” NTF for Parkinson’s disease (PD) and clinical trial thereof. We shortly summarize clinical trials of four NTFs, glial cell line-derived neurotrophic factor (GDNF), neurturin (NRTN), platelet-derived growth factor (PDGF-BB), and cerebral dopamine neurotrophic factor (CDNF), that were tested in PD patients, focusing mainly on GDNF and CDNF. We summarize current possibilities of NP-mediated delivery of NTFs to the brain and discuss whether NPs have impact in improving the properties of NTFs and delivery across the BBB. Emerging delivery approaches and future directions of NTF-based nanomedicine are also discussed.

## Introduction

### Potential of Neurotrophic Factors in Neurodegenerative Diseases and Open Challenges

Neurotrophic factors (NTFs) (trophic = survival) are typically proteins of 10 to 35 kDa in molecular weight that support the development, differentiation, survival and plasticity of neurons (Huttunen and Saarma, 2019). NTF-based therapies hold great promise for curing neurodegenerative diseases such as Alzheimer’s disease (AD), Parkinson’s disease (PD), amyotrophic lateral sclerosis (ALS), and Huntington’s disease (HD) that are currently incurable. Compared to potential alternative therapies, NTFs have the following advantages:

(a) NTFs are disease-modifying. All commercial and most of the emerging therapeutic approaches alleviate the symptoms of neurodegenerative diseases without curing them, while NTFs are able to slow down neurodegeneration and reverse the diseases at least in certain animal models and in some clinical trials (Lu et al., 2013; Chmielarz and Saarma, 2020).
(b) Decrease of NTF levels or knockdown of their receptors results in neuronal loss and other disease-related outcomes. Decreased levels of NTFs have been found in the brains of AD, HD, and PD patients (Rinne et al., 1989; Lorigados Pedre et al., 2002), while supplementation of NTFs can protect affected neurons in the animal models of neurodegenerative diseases. In mice deficient in NTFs or their receptors disease-related neurons are often lost or affected (Kramer et al., 2007; Lindahl et al., 2020).
(c) NTFs regulate the functional activity of neurons. This is achieved by regeneration of neuronal axons, stabilization and stimulation of synapses and regulation of the synthesis and release of neurotransmitters and expression of their transporters (Kumar et al., 2015; Castrén and Antila, 2017).

Despite the mentioned advantages that most of other potential therapies do not have, early studies have pointed out several problems associated with the clinical implementation of NTFs. Two of the most important obstacles observed are the side effects of NTFs and their low clinical benefit, whereas the main proposed reason for both is inadequate dosing and delivery of NTFs into the brain. The very first studies employed systemic administration of NTFs (Bartus and Johnson, 2017). However, most of the NTFs have short *in vivo* half-lives, poor pharmacokinetic properties and very low penetration rates through the blood–brain barrier (BBB). Thus, their access to the neuronal targets is restricted by their proteolytic degradation, different clearance mechanisms (such as rapid excretion by kidneys) and binding by various components of peripheral tissues (Thorne and Frey, 2001).

As the result, NTFs are currently delivered directly into the brain of PD patients using costly and risky intracranial surgery (Huttunen and Saarma, 2019). Only mid- and late-stage patients can be treated using brain surgery, since invasive treatments are considered unethical for the early stage patients. There is a great need for the efficient NTF peripheral delivery systems that could avoid intracranial surgery and allow treating early stage patients.

Current review focuses on the potential of nanoparticle (NPs)-based strategies to improve the stability and delivery of NTFs into the brain. We focus mainly on the delivery of NTFs in PD, and cover well-known NTFs as well as a new family of unconventional NTFs consisting of mesencephalic astrocyte-derived neurotrophic factor (MANF) and cerebral dopamine neurotrophic factor (CDNF) that have recently shown considerable therapeutic potential in the animal models of PD.

### Clinical Potential of NTFs in Parkinson’s Disease

**FIGURE 1:**
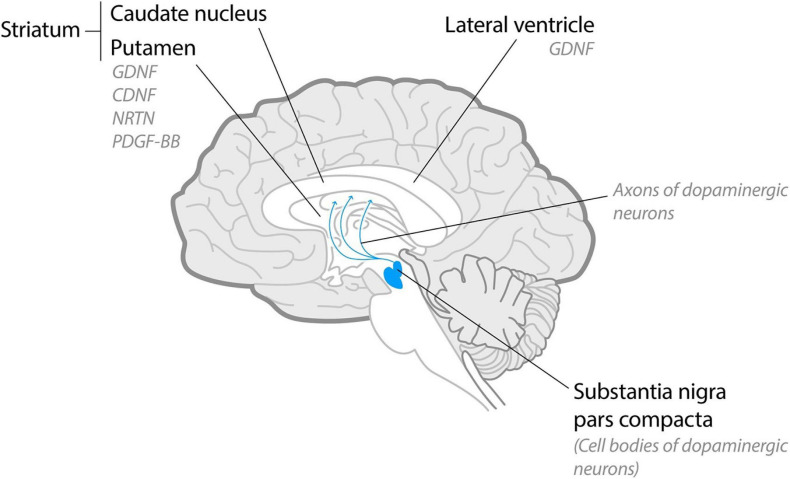
Schematic midsagittal cross-section of the human brain. Dopaminergic neurons localize in substantia nigra pars compacta and project their axons into striatum. In clinical trials with PD patients NTFs were delivered intracranially into lateral ventricles (glial cell line-derived neurotrophic factor, GDNF) or, predominantly, into putamen [GDNF, neurturin (NRTN), platelet-derived growth factor (PDGF-BB) and cerebral dopamine neurotrophic factor (CDNF)].

Parkinson’s disease is the second most common neurodegenerative disease that affects over 10 million people throughout the world. PD is characterized by progressive loss of nigrostriatal dopaminergic neurons in the midbrain that leads to severe movement disorders (Figure 1) and loss of peripheral dopaminergic neurons that mediates non-motor symptoms. Current PD treatment strategies are mostly based on dopamine replacement medicines and deep brain stimulation that temporarily alleviate the symptoms. However, none of the available treatments provide long-lasting relief from the symptoms and do not slow down or stop neurodegeneration. The use of NTFs as the disease-modifying treatment in PD has been explored for around 20 years, whereas several NTFs have shown a considerable potential (Table 1).

**TABLE 1:** Neurotrophic factors (NTFs) used in PD patients.

NTF	Characterization
Nerve growth factor (NGF)	The first NTF discovered (Levi-Montalcini and Hamburger, 1951). NGF was tested in one PD patient as a supporting tool for adrenal chromaffin tissue grafts (Olson et al., 1991).
Glial cell line-derived neurotrophic factor (GDNF)	Most studied NTF in PD. GDNF was discovered by Lin et al. (1993). In 1994 the first *in vivo* study with GDNF demonstrated that intranigrally injected GDNF improved apomorphine-induced rotational behavior in 6-hydroxydopamine (6-OHDA)-injected rats (Hoffer et al., 1994). Six clinical trials were conducted with GDNF (Gill et al., 2003; Nutt et al., 2003; Slevin et al., 2005; Lang et al., 2006; Heiss et al., 2019; Whone et al., 2019). In these trials, GDNF was injected into the brain in the form of protein or in the form of gene therapy with viral vector.
Neurturin (NRTN)	NRTN demonstrated neurorestorative properties in the nigrostriatal neurons in animal models of PD (Kotzbauer et al., 1996). Intraputaminal adeno-associated type-2 viral vector (AAV2)–delivered gene of NRTN was not superior to sham surgery when assessed using the UPDRS motor scores in clinical trials (Marks et al., 2008; Warren Olanow et al., 2015).
Platelet-derived growth factor BB (PDGF-BB)	PDGF-BB demonstrated neurorestorative properties in the nigrostriatal neurons in animal models of PD (Zachrisson et al., 2011). No change in clinical rating scores in placebo-controlled clinical trial with PD patients (Paul et al., 2015).
Cerebral dopamine neurotrophic factor (CDNF)	CDNF was discovered in 2007 and demonstrated neurorestorative properties in the nigrostriatal neurons in animal models (Lindholm et al., 2007). CDNF achieved its primary endpoint of safety and tolerability in a recent phase I-II clinical trial (Herantis Pharma, 2020; ClinicalTrials.gov: NCT03295786, NCT03775538).

Nerve growth factor (NGF) was the first NTF used in PD patients, and it was tested in one PD patient as support for the adrenal chromaffin tissue graft in the putamen (Olson et al., 1991). In the above-mentioned study, intraputamenal infusion of NGF was applied and resulted in the prolonged functional improvement compared to the earlier studies using the grafts without the support of NGF. However, later studies using the intraventricular infusion of NGF in AD patients concluded that the “negative side effects appear to outweigh the positive effects” (Jönhagen et al., 1998), at least for the intraventricular route of administration, and the clinical trials with NGF in PD patients were not conducted. In addition, dopamine neurons do not express a transmembrane receptor tyrosine-kinase (TrkA), a high-affinity receptor of NGF, and do not respond to NGF (Holtzman et al., 1995). In addition to NGF that was tested in one patient, four growth factors, glial cell line-derived neurotrophic factor (GDNF), neurturin (NRTN), platelet-derived growth factor (PDGF-BB), and CDNF have been tested in clinical trials in PD patients. The results of the clinical studies with GDNF, NRTN, and PDGF-BB were summarized by Sullivan and Toulouse (2011), recently updated by Huttunen and Saarma (2019), Barker et al. (2020), and Sidorova and Saarma (2020) and briefly summarized in Table 1. The clinical benefit of NTFs in these clinical trials was mostly assessed using Unified Parkinson’s Disease Rating Scale (UPDRS) demonstrating the severity of motor symptoms. Additionally, most of the studies evaluated the activity of dopamine transporters (DAT) using DAT positron emission tomography (DAT PET) as an indirect method for assessing the severity of the degradation of nigrostriatal dopamine neurons. While some of the studies demonstrated beneficial effects of NTFs, especially on DAT activity level, the others concluded a lack of the benefit. We discuss possible reasons of this discrepancy below.

**FIGURE 2:**
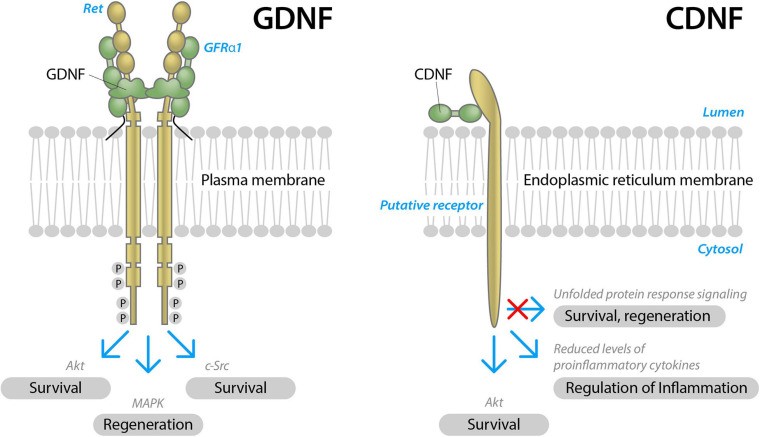
Schematic structure of two neurotrophic factors, GDNF and CDNF, and their modes of action in neurons. GDNF signals through receptor tyrosine kinase RET in the presence of a GDNF binding co-receptor, GFRα1. Upon activation, RET is transphosphorylated at cytoplasmic tyrosine residues and triggers complex intracellular signaling cascades, Akt, MAPK, and c-Src signaling pathways, promoting the survival and regeneration of neurons (modified from Airaksinen and Saarma, 2002). CDNF receptors are not identifyed yet. Presumably, CDNF signals through the receptor localized on the endoplasmatic reticulum membrane, alleviating unfolded protein response, reducing inflammation and promoting the survival of neurons.

Glial cell line-derived neurotrophic factor is the most studied NTF in PD. GDNF acts on neurons through its receptor, tyrosine kinase rearranged during transfection (RET) in the presence of a co-receptor, GDNF Family Receptor α1 (GFRα1) (Figure 2). RET activation triggers complex intracellular signaling cascades, promoting the survival and regeneration of neurons (Airaksinen and Saarma, 2002).

Six clinical trials in PD patients with GDNF were conducted. Two open-label trials demonstrated the improvement in UPDRS (Gill et al., 2003; Slevin et al., 2005), whereas one open-label and two double-blind placebo controlled clinical trials resulted in no improvement in UPDRS (Nutt et al., 2003; Lang et al., 2006; Whone et al., 2019). One gene therapy approach using an AAV2 vector to deliver GDNF gene to putamen of PD patients with some encouraging preliminary results is still ongoing: NCT01621581 (Heiss et al., 2019). Possible reasons of conflicting results from different clinical trials with GDNF can be attributed to several factors: delivery site of GDNF in the brain (putamen vs. cerebral ventricles), origin of the NTF (mammalian vs. bacterial), development of anti-NTF antibodies, different NTF dosage/delivery regimens, and differences in the age of the patients and stages of the disease. Below we briefly comment on the studies, where GDNF did not show improvement in UPDRS compared to placebo.

In the first clinical trial, GDNF was delivered into lateral ventricle (Nutt et al., 2003). It is currently suggested that GDNF did not reach nigral neurons using this delivery method. In the study by Lang et al. (2006), development of anti-GDNF antibodies in the blood of PD patients was observed, suggesting the leakage of GDNF into periphery and, as a result, its insufficient delivery of NTF to the brain that might have compromised the results. Indeed, only less than 10% total putaminal coverage by GDNF was observed in this clinical trial, suggesting insufficient delivery (Salvatore et al., 2006). In the study by Whone et al. (2019), during a course of 40 weeks, intermittent GDNF intraputamenal infusions (120 μg GDNF to each putamen) were performed once every 4 weeks. The cumulative 4-week GDNF dose per putamen in this study was 3.5-fold smaller than in the continuous dosing study by Lang et al. (2006) (120 μg vs. 420 μg). The resulting tissue GDNF concentrations in the exposed volumes were estimated to be ∼18-fold lower in the study by Whone et al. (2019), suggesting that delivered GDNF dose was insufficient. Altogether, three clinical trials, where GDNF did not demonstrate improvement in UPDRS compared to placebo, had considerable drawbacks in the study design that have been widely discussed. In addition, all these studies used GDNF produced in *Escherichia coli* that rendered unglycosylated GDNF. GDNF has seven S-S-bridges that are not formed in *E. coli*. For these reasons bacterially produced GDNF was shown to possess a lower stability and activity than the GDNF produced in mammalian cells (Piccinini et al., 2013, Grondin et al., 2019).

Cerebral dopamine neurotrophic factor and MANF are the newest discovered NTFs with unconventional structure and modes of actions (Lindahl et al., 2017; Figure 2). CDNF was discovered in 2007 and showed a greater potential to cure PD *in vivo* compared to the “gold standard” NTF–GDNF (Lindholm et al., 2007; Voutilainen et al., 2011). A first-in-man clinical study with intraputamenal CDNF, a Phase I-II randomized, placebo-controlled, double-blind, multicenter clinical study in patients with advanced PD (>10 years since first motor symptoms) was conducted by Herantis Pharma Plc. and was completed in August 2020 (manuscript in preparation). CDNF was administered into the putamen of the patients once a month for 6 months at either mid-dose (120 μg for 2 months and 400 μg for the following 4 months) or high-dose (120 μg for 2 months, 400 μg for 2 months and 1200 μg for 2 months), followed by a 6-month active treatment extension study, in which all patients received one of two doses of CDNF, including the placebo group. CDNF achieved its primary endpoint of safety and tolerability. In addition to the primary safety endpoint, secondary endpoints assessing e.g., severity of motor symptoms by UPDRS, and exploratory assessments evaluating the integrity of nigrostriatal dopaminergic system by DAT PET, actigraphy and cerebrospinal fluid proteomics were included. Significant increases in DAT PET signaling and improved UPDRS scores were observed in some but not all CDNF-treated patients. Further clinical studies with non-invasive CDNF delivery are planned (Herantis Pharma, 2020).

As mentioned, a sub-group of patients in the clinical study with CDNF and all GDNF-treated patients in a study with GDNF conducted by Whone et al. (2019) had a significant increase in DAT PET signaling, but only some of the patients had improved UPDRS score: *a post hoc* analysis found nine (43%) patients in the GDNF-treated group but no placebo patients with a large clinically important motor improvement (≥10 points) in the OFF state. Whone et al. (2019) thoroughly discussed possible reasons for the phenomenon and asked whether patient phenotypes had played a role. However, *post hoc* covariate analyses investigating phenotypic characteristics such as age, disease duration, disease severity, and tremor predominance did not identify any subtype of the patients who experienced an enhanced benefit (Whone et al., 2019). It would be tempting to identify a subgroup of PD patients that are either more or less likely to respond to GDNF/CDNF therapy.

Together with CDNF, MANF belongs to a novel evolutionarily conserved family of NTFs. MANF was discovered in 2003 and was shown to selectively protect nigral dopamine neurons *in vitro* (Petrova et al., 2003) and *in vivo* in animal models (Voutilainen et al., 2009). Despite that MANF was discovered earlier than CDNF, it has not yet been tested in clinical trials. The potency of MANF to protect dopamine neurons *in vivo* in a rat PD model (unilateral intrastriatal injection of 6-OHDA) was shown to be lower compared to CDNF. While MANF demonstrated potential to rescue cell bodies of dopamine neurons in the *substantia nigra (SN) pars compacta* (SNpc) in a 6-OHDA rat model, its protective effect on neuronal axons (the fibers of dopamine neurons in the striatum) was modest or absent (Voutilainen et al., 2009, 2011), making it a dubious NTF candidate for clinical trials in PD patients. However, the fact that MANF mitigates the inflammatory response and ER stress warrants further studies of neurorestorative potential of this NTF in other neurodegenerative diseases (Kovaleva et al., 2020; Yagi et al., 2020).

## Discussion

### Ideal NTF and Hypothetical Perfectly Designed Clinical Trial

Clinical studies with NTFs have vastly advanced our understanding of the proteins that should be taken into account for the design of clinical trials with NTFs in PD patients. Some of the design parameters are shown in Box 1.

**BOX 1. Properties of the ideal NTF for PD.**

1. *Can be delivered systemically (e.g., intranasal or injection enabling to avoid brain surgery);*
2. *Good diffusion in the brain enabling to reach target cells;*
3. *Stability in the body fluids (e.g., blood) and tissues enabling to reach the target cells in sufficient concentrations;*
4. *Specificity toward dopamine neurons enabling reduction of side effects;*
5. *Ability to treat non-motor symptoms;*
6. *Systemic delivery without side effects and immune response;*
7. *Possibility of starting treatment soon after diagnosis when significant number of dopamine neurons are still alive.*

#### Delivery Method–Systemic vs. Intracranial

Systemic (non-invasive) administration of NTFs would be the ideal method of delivery. Although early research articles demonstrated some benefits of NTFs delivered systemically, later better controlled studies failed to replicate the efficiency or identified serious side effects of NTFs that limited the dosage (Bartus and Johnson, 2017). These disappointing results promoted the implementation of the invasive methods, where NTFs were delivered directly into the brain, eihter by infusion of the protein using mini-pumps or gene delivery with viral vectors. There are several serious problems associated with the intracranial delivery. Firstly, intracranial delivery implies a costly and risky neurosurgical procedures associated with safety concerns that may result in different adverse outcomes such as inflammation (Lang et al., 2006). Secondly, development of anti-NTF antibodies may occur, if NTF leaks into the periphery. Previous study suggested that anti-GDNF antibodies developed due to the leakage of intra-putaminally infused GDNF into the periphery (Barker et al., 2020). Thirdly, intracranial delivery of NTFs does not allow treatment of many non-motor symptoms of PD. Finally, due to the ethical considerations and severity of microsurgical intervention, only middle to late-stage patients are eligble for the intracranial treatment with NTFs that poses a problem for efficacy of NTFs: the more advanced is PD, the less DA neurons are in the brain to protect leading to decreased efficiency of the therapy.

#### Delivery Site, Dosing Device and Diffusion Inside the Brain

Degeneration of dopamine neurons starts from their synapses and the axons in caudate putamen (Figure 1), whereas cell bodies in the SNpc degenerate later. NTFs are not able to reverse the apoptosis of completely degenerated neurons; instead, they restore the axons of the stressed and degenerating dopamine neurons and their contacts (Sidorova and Saarma, 2020). Thus, the degenerating axons in caudate putamen are the main targets of NTFs, and according to current understanding, the NTFs should be delivered to the axons of the neurons into caudate putamen (discussed in more details below).

There are mainly two methods to deliver NTFs directly into the brain:

(1) The intracerebroventricular method that relies on the implantation of a catheter and or an internal reservoir with the drug followed by controlled drug injection from this reservoir into lateral ventricle.
(2) The intraparenchymal/intracerebral method relying on the placement of tiny catheters into the brain parenchyma and continuous or periodical connection of the catheters to the external drug reservoir. Drug either diffuses inside the brain by a concentration gradient (“regular” injection) or “pumped” using a positive pressure gradient pump [convection enhanced delivery (CED)] (Furtado et al., 2018).

In the first attempt to treat human PD patients with GDNF, the intracerebroventricular delivery method was applied and resulted in no improvement in the UPDRS (Nutt et al., 2003). Since GDNF diffuses poorly in the brain, it was hypothesized that GDNF never reached the nigral target neurons in sufficient quantities. Since the pool of cerebrospinal fluid in the brain ventricles is turned over every 4–5 h and any drug injected into the ventricles is flushed into the blood with very limited BBB penetration (Furtado et al., 2018), it is reasonable to hypothesize that intraventricular NTF delivery is a suboptimal delivery method for most of the NTFs in PD. In the second phase II trial 15 μg/24 h of GDNF was continuously infused to the putamen of the patients. The treatment was rather well tolerated, yet showed no therapeutic benefit for the patients (Lang et al., 2006). Importantly, the development of GDNF-neutralizing antibodies in six patients argues for the peripheral leakage of GDNF, suggesting problems with the mini-pump connection and uncertainties in the amounts of GDNF delivered into the brain. In the third double-blind phase II clinical study in PD patients intraputamenal CED of GDNF protein was used, where GDNF was delivered once a month to the putamen at a dose of 120 μg per putamen. Again, the motor UPDRS scores did not significantly differ between the GDNF and the placebo groups, albeit significant improvements were observed in individual cases. It was suggested that intermittent intraputamenal CED of GDNF resulted in an adequate coverage of the putamen with NTF, overcoming prior drug delivery limitations (Whone et al., 2019). Moreover, since GDNF binds readily to the cell surface and extracellular matrix heparin sulfate proteoglycans (Bespalov et al., 2011) and diffuses poorly in the brain, it was suggested that 120 μg per putamen per month was perhaps not enough to induce a significant effect. The coverage of GDNF delivered to the putamen utilizing CED is around 30% (transfrontal approach) or 50% (posterior approach) (Barker et al., 2020). For comparison, the coverage of the putamen in the CDNF Phase I trial was predicted to be around 60–75% using similar device and delivery site (e.g., infusion into putamen using CED). Thus, it is expected that bioavailability of CDNF to the neuronal axons is significantly higher compared to GDNF due to its better diffusion properties (Voutilainen et al., 2011).

#### NTF Dosage and Continuity of the Infusion–Continuous vs. Intermittent

There is still a debate regarding the NTF doses and continuity of infusion to achieve the best results. The majority of clinical trials in PD patients have been conducted using continuous infusion of high doses of NTFs or their constitutive overexpression from viral vectors. However, by characterizing the distribution, toxicity and the functional effects of GDNF after CED into the striatum of rats *in vivo*, Taylor et al. (2013) concluded that high concentrations (above 0.6 μg/μl) of GDNF were associated with neuronal and synaptic toxicity. This study advised that “CED of low concentrations of GDNF, with dosing intervals determined by tissue clearance, has most potential for effective clinical translation by optimizing distribution and minimizing the risk of toxic accumulation.” Interestingly, the preliminary report on phase I-II clinical study of CDNF in PD patients also suggested that the improvement of the DAT PET was mostly characteristic for the mid-dose CDNF patients (120 μg for 3 months + 400 μg for 3 months) compared to the high-dose patients (120 μg for 2 months + 400 μg for 2 months + 1200 μg for 2 months) (Herantis Pharma, 2020). Considering that cellular responses to the NTFs can be bell-shaped [e.g., effect of the GDNF on the neurite outgrowth is higher in case of lower doses (Mills et al., 2007)], it is crucial to optimize the dose of each NTF in clinical trials individually, depending on the delivery method as well as the physico-chemical and biological characteristics of the NTF.

#### Patient Age and Stage of the Disease

When PD is first diagnosed, there is around 50–70% reduction in the density of axons of dopamine neurons in the putamen and about 30% reduction in the number of cell bodies in the SNpc (Cheng et al., 2010). By 5–6 years post-diagnosis, the putamen is nearly completely devoid of the axons of dopamine neurons, leaving only a small number of neuronal axons that can be restored by potential treatments (Kordower et al., 2013). Currently, only middle- to late-stage (mainly >8 years post-diagnosis) PD patients are eligble for the intracranial treatment with NTFs, meaning there are almost no axons to restore in the putamen. In the ideal case, the treatment of PD patients should be started as soon as the diagnosis is clear and confirmed (some time windows are advised to follow the PD progression, since there are curently no 100% reliable methods for early diagnosis), and this is ethically acceptable only in case peripheral delivery. Development of novel (targeted) delivery systems would help to overcome this problem and allow treatment of PD patients with NTFs immediately after the diagnosis.

### Solving the Delivery Problem: From Intracranial Delivery to Nanoparticles and Brain Targeting

Particle-based delivery systems are of considerable interest because particles can improve the solubility of NTFs, prolong the half-life of NTFs in blood circulation and/or in the brain, provide a controlled sustained release of NTFs and improve the local delivery of NTFs or enable the delivery in a systemic manner (Tan et al., 2012). For PD therapy, particles can be delivered to the brain by at least three different routes: locally, systemically or intranasally, and by the means of differently sized particles: microparticles or NPs (Torres-Ortega et al., 2019). Early proof of concept studies on NTF delivery with particles focused mainly on microparticles and local administration *via* stereotactic surgery (Jollivet et al., 2004; Clavreul et al., 2006; Tatard et al., 2007; Garbayo et al., 2011, 2016; Herrán et al., 2013, 2014; Requejo et al., 2015). As an important milestone, the safety and efficiency of GDNF encapsulated into microparticles was demonstrated in the experiments with parkinsonian monkeys. In this study micro-encapsulated GDNF injected into putamen provided motor improvement and dopaminergic function restoration in monkeys lesioned by 1-methyl-4-phenyl-1,2,3,6-tetrahydropyridine (MPTP) (Garbayo et al., 2016). In another set of studies, superior restorative effect of the combination of GDNF and vascular endothelial growth factor (VEGF) encapsulated into micro- or nanospheres and implanted into 6-OHDA-lesioned rats was demonstrated (Herrán et al., 2013, 2014).

Increasingly, the local delivery by microparticles was replaced by systemic delivery using particles with at least one size dimension below 100 nm, e.g., NPs. Similarly to microparticles, NPs improve the pharmacokinetic properties of NTFs, and due to their small size they also facilitate the delivery of NTFs through the BBB. NPs or other nanoscale structures that are a part of drug formulations, are referred to as nanomedicines. Box 2 defines the use of terms “NPs” and “nanomedicines” in the current review.

**BOX 2. Definitions of nanoparticles and nanomedicines.**

***Nanoparticles:*** *Natural, incidental or manufactured particles with three external dimensions at* “*nanoscale*,” *i.e., in the size range of 1–100 nm* (ISO/TS 80004-2:2015). *In the current review we mean manufactured (synthetic) nanoparticles.*

***Nanomedicine:*** *“A branch of medicine that applies the knowledge and tools of nanotechnology to the prevention and treatment of disease. Nanomedicine involves the use of nanoscale materials /…/ for diagnosis, delivery, sensing or actuation purposes in a living organism” (Nature, 2020). Colloquially, the plural form “nanomedicines” usually refers to the products of nanomedicine.*

Within the realm of nanomedicine, NPs are often used as non-viral vectors for drug delivery to different cells and organs, including the brain. Depending on the properties of the drugs, they can be either placed into NPs or attached to their surfaces. The first NPs for drug delivery were developed in the 1960s (Kreuter, 2007). Despite the relatively long history of nanomedicine, however, there were only 56 U.S. Food and Drug Administration (FDA)-approved nanomedicines on the market in 2016 (Bobo et al., 2016) and around 30 nanomedicines approved by the European Medicines Agency (EMA) in 2018 (Soares et al., 2018). However, the number of approved medicines is growing rapidly, and more than dozen of nanomedicines are currently in clinical trials (Gadekar et al., 2021). Remarkably, one of the last approved nanomedicines are lipid-based NPs that are now a key component of COVID-19 mRNA-based vaccines, BioNTech/Pfizer’s BNT162b2 and Moderna’s mRNA-1273 (Shin et al., 2020; Nature Reviews Material Editorial, 2021), demonstrating increasing importance of nanomedicines.

Most of the approved nanomedicines are anti-cancer drugs, whereas the main benefit of “nano” is in the improvement of the physico-chemical properties and stability of the active drug and its passive accumulation in tumor cells, while the active cell- and organ-targeting has not been achived yet, despite some encouraging data from animal models. In the highly cited and discussed article, after analyzing the scientific literature from the past 10 years, Wilhelm et al. (2016) concluded that “only around 0.7% (median) of the administered NP dose is found to be delivered to a solid tumor.” This problem is more than relevant for the brain targeting by NPs, since brain is the most poorly accessible organ in the body due to the presence of the BBB that protects the brain from harmful environmental factors, chemicals and drugs.

To the best of our knowledge, there are no FDA-approved nanomedicines on the market yet that facilitate the penetration of drugs through the BBB. However, there are numerous research articles demonstrating that drugs that do not cross the BBB can be loaded into NPs and transported into the brain *in vivo* (Saraiva et al., 2016). In the current review, we focus on NP-mediated delivery to the brain, although many alternative non-invasive strategies are being actively explored to tackle or surmount the BBB, including several relatively new approaches such as transient opening of the BBB by ultrasound, laser light or chemicals, intranasal delivery, or facial intradermal injections (Khan et al., 2017; Furtado et al., 2018). Due to the small size and large surface area, NPs display advantages as drug delivery systems, posing high drug loading capacity and penetration of biological barriers. As exemplified below, in some cases NP-mediated delivery can be combined with alternative approaches, such as transient opening of the BBB and intranasal delivery. In addition, NPs can be functionalized with brain-targeting ligands to reinforce their BBB penetration.

**FIGURE 3:**
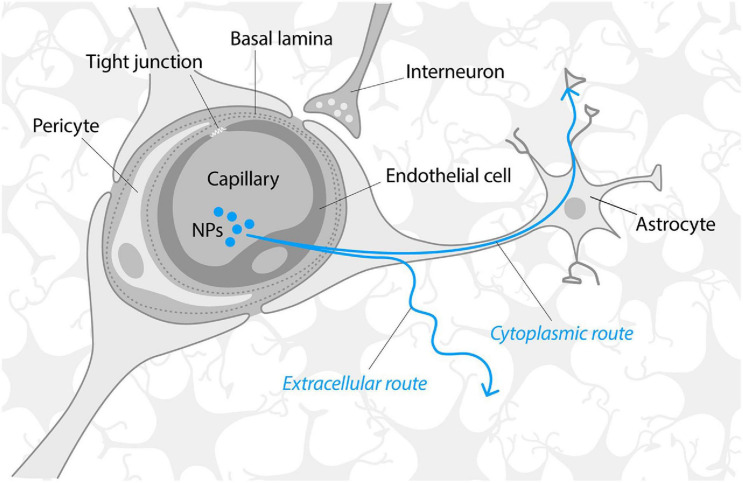
Schematic structure of the blood–brain barrier. Modified from Abbott et al. (2006) and Lau et al. (2013). Neurotrophic factors and nanoparticles are mainly transported through the BBB via receptor-mediated transcytosis or endocytosis. Once across the BBB, NTFs, and NPs must traverse the extracellular space (ca 40 nm microenvironment formed by gaps between brain cells) that limits the diffusion of molecules that are significantly larger than 40 nm or/and bind to the extracellular matrix. It has been suggested that NPs may also follow the alternative, “speedy cytoplasmic route” through the brain tissue, assumingly via the connections of astrocytes (Kreuter, 2014).

The BBB consists of tightly connected monolayer of brain endothelial cells sheathed by astrocyte end-feet (Figure 3) that is impermeable to almost 100% of large molecules and about 95% small molecules. The BBB acts as a physical barrier due to the presence of intramembranous proteins called tight junctions that connect endothelial cells and form a highly selective network to force most molecular traffic toward a transcellular route (i.e., through cells) across the BBB, rather than the paracellular route (i.e., through the intercellular space between cells), as in most endothelia (Abbott et al., 2006). BBB permeability is actively regulated by the cross-talk between endothelial cells, astrocytes and several other cell types (microglia, pericytes, oligodendrocytes, neurons etc.) that localize on the “brain” side and, altogether, form a functional structure known as the neurovascular unit (Furtado et al., 2018).

The composition, characteristics, permeability regulation of the BBB and their considerations for NP-mediated delivery have recently been described in an excellent review by Furtado et al. (2018). Proteins such as NTFs are transported through the BBB *via* receptor-mediated transcytosis (Sweeney et al., 2018), e.g., they are taken up by endocytosis of endothelial cells on the “blood” side and released to the “brain” side. The surface of the capillary basement membrane is almost completely covered by the end-feet of astrocytes that are separated from the capillary endothelium by a distance of only 20 nm (Sonavane et al., 2008). Thus, to reach the brain, the molecules, NTFs of NPs should be transported across the membranes of several cell types, i.e., endothelial cells and astrocytes. Once across the BBB, molecules or NPs must traverse extracellular space (narrow microenvironment formed by gaps between brain cells) that is around 40 nm and is partly filled by the dense negatively charged extracellular matrix (Nicholson and Hrabìtová, 2017). Extracellular matrix limits the diffusion of the molecules that bind to the extracellular matrix (such as GDNF, NRTN) and the transportation of NPs significantly bigger than 40 nm due to size limitations. However, it has been suggested that once in the brain, NPs may follow the speedy cytoplasmic route through brain tissue, whereas astrocytes may provide such a cytoplasmic route (Kreuter, 2014).

As a rule, only a tiny fraction of NTFs introduced by peripheral delivery methods reaches the brain, since NTFs are rapidly inactivated by cellular and extracellular proteases, excreted by kidneys and bound by proteins and other components of the blood and tissues (Thorne and Frey, 2001). When placed inside or attached to the NPs, NTFs are stabilized from enzymatic clearance and sequestration. The efficiency of NPs in delivering the drug to the brain mainly depends on the circulation times of NPs in the blood and the ability to cross the BBB that are largely defined by the NP size, composition and, especially, surface functional groups.

#### Nanoparticle Size

*In vitro* studies conducted with different cell lines and various types of NPs have suggested that the most optimal cell association and endocytotic uptake occur in the NP size range of 15–70 nm (Jiang et al., 2008; Furtado et al., 2018). In general, while >100 nm NPs are less efficient in the BBB penetration, <5 nm NPs are quickly removed from systemic circulation by renal clearance and will not reach brain in sufficient quantities. Shilo et al. (2015) studied the uptake of 20, 50, 70, and 110 nm gold (Au) NPs to mouse immortalized brain endothelial cell line bEnd3 *in vitro* and showed that the 70 nm Au NPs were the most optimal for uptake while the 20 nm Au NPs possessed the maximum free surface area for uptake by bEnd3 cells. In another study, Ohta et al. (2020) investigated the penetration of 3, 15, and 120 nm Au NPs through the monolayer of bEnd3 cells *in vitro*, treated shortly by focused ultrasound to induce transient widening of their tight junctions. In this experiment, the 15 nm Au NPs showed the highest passage through the cell monolayer. However, *in vitro* studies do not provide information on the fate of NPs inside the brain, e.g., their distribution among the brain regions, cell population and clearance mechanisms. Sonavane et al. (2008) studied the tissue distribution of 15, 50, 100, and 200 nm Au NPs after i.v. administration in mice, and found that the 50 nm and especially the 15 nm NPs were the most widespread in all organs, including the brain. The NP percent in brain compared to the delivered dose was low for all NPs: 0.075% for the 15 nm, 0.036% for the 50 nm, 0.023% for the 100 nm, and 0.0003% for the 200 nm NPs, demonstrating that smaller NPs should be preferred for the brain delivery. More well-designed *in vitro* transcytosis studies, and especially relevant *in vivo* studies are crucially needed to understand NP size-dependent BBB penetration and the later fate of NPs in the brain.

#### Nanoparticle Composition

**FIGURE 4:**
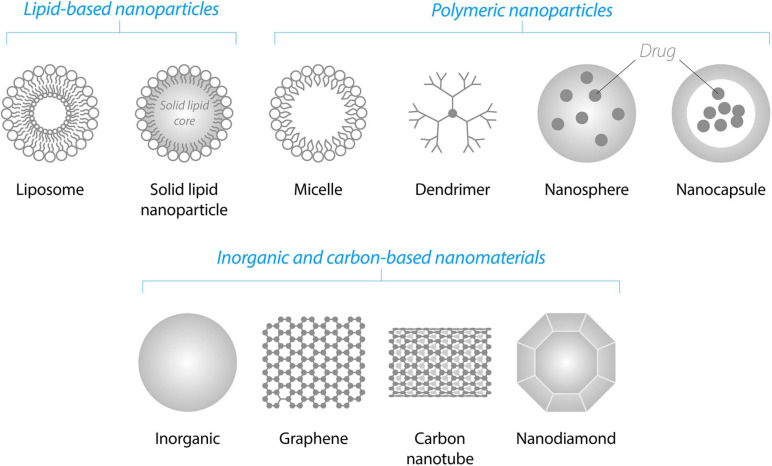
Schematic illustration of various classes of NPs suitable for the delivery of NTFs through the blood–brain barrier.

There are many classes of NPs and a few dozen of them are already on the market as a part of various nanomedicines (Bobo et al., 2016). Below we briefly highlight the NP classes that we consider the most promising for the NTF delivery to the brain (Figure 4).

Lipid-based NPs are liposomes and solid lipid NPs. Liposomes are among the simplest and most commonly used biodegradable NPs enabling the delivery of the drugs through the BBB (Torchilin, 2005; Wohlfart et al., 2012). They consist of a lipid bilayer(s) that form a hydrophilic core. Due to this amphiphilic structure, liposomes are able to incorporate both hydrophilic and hydrophobic drugs. Hydrophilic drugs may be entrapped inside the hydrophilic core of the liposomes and hydrophobic drugs may be placed into the internal hydrophobic part of the lipid bilayer(s). Solid lipid NPs possess a solid hydrophobic core consisting of biocompatible lipids such as fatty acids, waxes or triglycerides stabilized by surfactants. These NPs are biocompatible, possess high drug entrapment efficiency, stability and controlled release (Furtado et al., 2018).

Polymeric NPs are dendrimers, nanocapsules, or nanospheres consisting of synthetic or natural polymers. Some of the most widely employed polymers are poly-n-butyl cyanoacrylate (PBCA), poly(lactic-co-glycolic acid) (PLGA), poly(glycolic acid) (PGA), poly(L-glutamic acid) (PLE), poly(lactic acid) (PLA), human serum albumin, and chitosan. One of the first polymeric NPs for NTF delivery to the brain was PBCA NPs, which have been increasingly replaced by biodegradable PLA, PLE, or PLGA NPs. PLGA NPs have been approved by FDA and EMA for use in drug delivery systems (Kurakhmaeva et al., 2009; Danhier et al., 2012).

Dendrimers, such as polyamidoamine dendrimers (PAMAMs), have been successfully used for the delivery of NTF genes. Under physiological conditions, PAMAM-DNA complexes have a positive net charge that facilitates their interaction with net negatively charged cell membrane.

Inorganic non-degradable NPs such as fullerenes, metal-based NPs and carbon-based nanomaterials have a lower clinical potential compared to biodegradable NPs. However, inorganic NPs are frequently used as tools in the proof-of-concept studies, since they are often easily detectable in cells by analytical methods: inductively coupled plasma mass spectrometry for the detection of metals, measurement of surface plasmon resonance characteristics for Au and Ag NPs, and fluorescence characteristics for graphene quantum dots, etc. In addition, several exciting articles have recently demonstrated that some inorganic NPs such as Au and graphene quantum dots efficiently penetrated the BBB and *per se* prevented and reversed alpha-synuclein (αSyn) aggregation in a PD model [graphene quantum dots (Kim et al., 2018)], and inhibited amyloid beta (Aβ)-related toxicity in AD models [Au NPs (Javed et al., 2019)] and carbon dots (Zhou et al., 2019). In the section “Perspective” of this review, we discuss in more detail potential therapeutic impact of these NPs.

**FIGURE 5:**
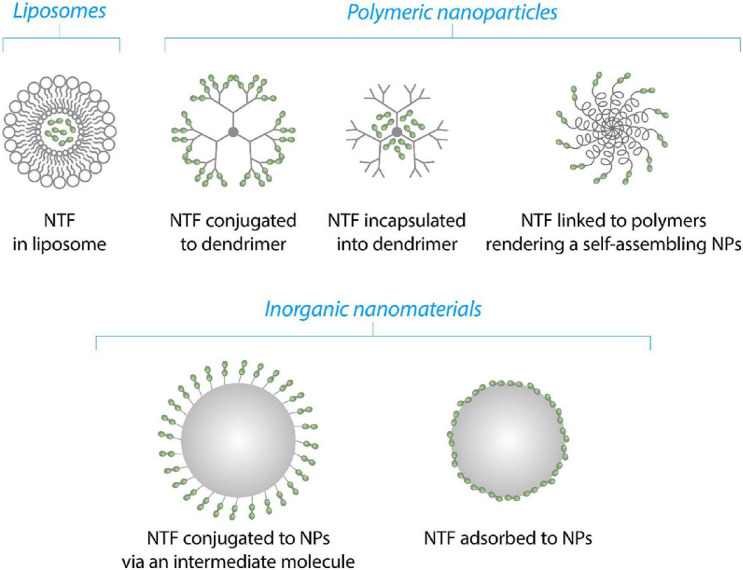
Schematic illustration of the mechanisms of NTFs’ (green dots) binding to NPs.

**FIGURE 6:**
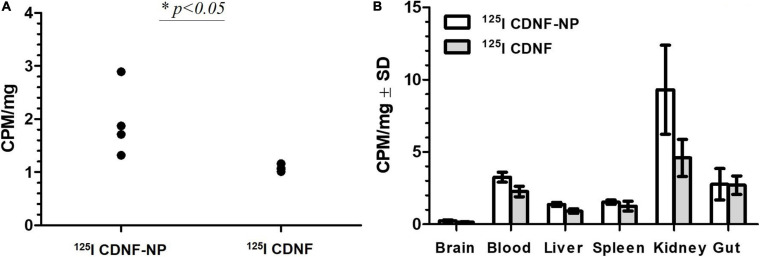
Delivery of ^125^I-labeled CDNF through the blood–brain barrier *in vivo* using nanoparticles [counts per minute (CPM) per mg tissue]. ^125^I CDNF was adsorbed on the surface of 200 nm poly(lactic-co-glycolic acid) nanoparticles (^125^I CDNF-NP) or used without NPs (^125^I CDNF) and administered subcutaneously *in vivo* in rats (100 μl equaling to 954 000 CPM were delivered). Rats were perfused with PBS for 20 min. **(A)** ^125^I CDNF in the brain after 1 h (CPM/mg brain). **(B)** Distribution of CDNF in different organs after 1 h (CPM per mg tissue).^∗^*p* < 0.05, *n* = 4. Unpublished data. Experiments were performed under the permits ESAVI/12830/2020.

Neurotrophic factors are encapsulated into NPs or attached to their surface using different methods, depending on the physico-chemical properties of NPs and NTF (Figure 5). NTFs or genes of NTFs may be incapsuated into PAMAMs or loaded into liposomes as described for BDNF and GDNF genes (Xing et al., 2016; Lin et al., 2020). In case of polymeric as well inorganic NPs, NTFs can be conjugated via surface groups or adsorbed passively onto the surface on NPs (this study, Figure 6). Finally, NTFs can be attached to the polymers of polymer-polyethylene glycol (PEG) complexes to form self-assembilng NPs (Jiang et al., 2018).

#### Nanoparticle Surface Modifications (Coatings)

Nanoparticle surface modifications include molecules, polymers or peptides covalently or non-covalently attached to the NPs. These surface ligands are crucial for improved delivery of NPs to the brain, since they either prolong the circulation of NPs in the blood or facilitate the penetration of NPs through the BBB, mostly *via* the interaction of NP-attached ligand with the receptors on brain endothelial cells.

##### Surface functionalizations prolong NP circulation in the blood

The first type of surface functionalization has been applied to “stealth” NPs–e.g., to increase their circulation rates in the bloodstream by protecting them from the adsorption of blood proteins that promote NP uptake by the cells of the reticuloendothelial system (Ke et al., 2017). In addition, blood proteins and other molecules that non-specifically adsorb onto the surface of NPs in biological fluids (e.g., blood) may reduce the efficiency of targeting ligands by physically masking the latter’s action (Monopoli et al., 2012). To prevent the adsorption of proteins, NP clearance by immune cells, and masking of targeting ligands, NPs are often functionalized with PEG or dextran. This is one of the oldest strategies for improving the circulation rates of NPs and individual drugs. In addition, dense coating of NPs with PEG improves their diffusion in the brain, even for NPs of >100 nm in size, whose limited diffusion inside the brain is otherwise minimal (Nance et al., 2012).

##### Non-covalent surface modifications improving BBB targeting

The second type of NP surface modification was described decades ago, which relies on the application of different surfactants such as polysorbate 80 or poloxamer 188 (PX188) that guide NPs to the brain (Gulyaev et al., 1999). The precise mechanism of surfactant-NP-mediated uptake through the BBB is still under debate, from increasing the adsorption of NPs to blood capillaries and increased retention until inhibition of the efflux system, especially by P-glycoprotein (Kreuter, 2014). One of the prevailing explanations suggests that polysorbate 80, PX188 and some other surfactants facilitate the adsorption of apolipoproteins E or A–I from blood that trigger the interaction of surfactant-coated NPs with receptors on brain endothelial cells, thereby enabling NPs to cross the BBB *via* endocytosis (Kreuter et al., 1997; Saraiva et al., 2016). In our study, we employed this strategy to deliver peripherally injected radioactively labeled CDNF through the BBB (Figure 6, our unpublished preliminary data). For that we adsorbed ^125^I-labeled CDNF to the non-toxic biodegradable PLGA NPs (200 nm, Phosphorex) and overcoated the construct with PX188 to enhance their brain targeting. We demonstrated that a 1.5-fold more radioactivity (expressed as counts *per* minute, CPM) was detected in the brain of rats in terms of the CDNF adsorbed to NPs (^125^I-CDNF-NP) compared to free non NP adsorbed CDNF (^125^I-CDNF) 1 h after subcutaneous injection. In addition to the radioactivity in the brain, we also measured radioactivity values in other organs as a proxy for CDNF distribution. We observed that: (1) the fraction of CDNF was negligible in the brain (0.03% without NPs and 0.046% with NPs) compared to other organs, and (2) in most organs, their radioactivity levels were increased in terms of CDNF-NP compared to free CDNF (Figure 6B). This suggests that the effect of NPs was not brain-specific: NPs rather increased the stability of CDNF, leading to increased levels of CDNF in different organs including the brain. To specifically target the brain, novel mode advanced methods are needed, and some approaches are discussed below.

##### Covalent surface modifications improving BBB targeting

The third and the most “modern” approach relies on the surface functionalization of NPs with BBB targeting ligands such as transferrin, insulin, lactoferrin, apolipoproteins, antibodies, cell penetrating peptides, or homing peptides. These ligands are either recognized by the respective receptors on brain endothelial cells, endocytosed by endothelial cells and released on the “brain side” of the BBB (transferrin, insulin, lactoferrin, apolipoproteins, antibodies, and many homing peptides), or penetrate directly through cellular membranes (cell-penetrating peptides) (Sullivan and Toulouse, 2011; Niewoehner et al., 2014). Some representative examples of targeting ligands used for the transportation of NTFs through the BBB or bypassing the BBB are shown in Table 2. One of the pioneering works in this field was published by Wu and Pardridge (1999). The authors conjugated BDNF to the transferrin receptor antibody OX26 and demonstrated restoration of CA1 region in hippocampus after ischemia in rats after i.v. delivery of PEG-BDNF-OX26. Later, transferrin and lactoferrin were attached to different types of nanocarriers and used to deliver various NTFs across the BBB (Table 2). However, this method provided only about a 2 to 3-fold improved transport across the BBB (Xing et al., 2016). PAMAMs functionalized with lactoferrin and PEG were used to deliver the GDNF gene in a 6-OHDA rat model. Since both lactoferrin and transferrin are large proteins (over 70 kDa), subsequent attempts were made to reduce the length of these targeting ligands to improve the efficiency of delivery. For example, Prades et al. (2012) conjugated short homing peptide THRPPMWSPVWP (THR) targeting the transferrin receptor to Au NPs, together with the therapeutic peptide CLPFFD to destroy the toxic aggregates of β-amyloid in AD. The authors showed that the concentration of Au NPs in rats was about 3–4 times higher than Au NPs without THR after 1 h i. p. injection. Still, the percentage of THR-CLPFFD-Au NPs was low in the brain: 0.0765% from injected dose in the best case, at 2 h after i. p. injection. Since it was previously demonstrated that short peptides called angiopeps exhibited a higher transcytosis capacity and parenchymal accumulation in an artificial *in vitro* BBB model than transferrin and lactoferrin (Demeule et al., 2008), angiopep- and PEG-conjugated dendrigraft poly-L-lysine NPs were used to transport GDNF gene through the BBB (Huang et al., 2013). Rotenone-treated rats with five injections of angiopep-PEG-dendrigraft poly-L-lysine NPs bearing GDNF gene demonstrated the best improved locomotor activity and an apparent recovery of dopamine neurons compared to the control and groups receiving lower doses of angiopep-PEG-dendrigraft poly-L-lysine NPs. The exact percentage of NPs reaching the brain was not determined in this study. However, the body distribution of ^125^I-labeled angiopep-conjugated NPs was studied by Ke et al. (2009) in murine brains 48 h after i.v. injection of NPs with different ratios of angiopep. This study demonstrated that the concentrations of ^125^I-labeled angiopep-conjugated NPs with different ratios of angiopep were 3.06, 5.12, and 8.42-fold higher in the brain than NPs without angiopep, where the increasing rate of brain uptake corresponded to the higher ratio of angiopep in NPs. Thus, short peptides seem to be a promising approach to increase NP delivery to the brain, and the research and development of new brain-targeting peptides or optimization of old ones may be a valuable strategy.

**TABLE 2:** Ligands used for nanoparticles, for the transportation of neurotrophic factors through the blood–brain barrier.

Strategy	Ligand	Example	Effect; administration route*	References
Targeting of transferrin receptor	TfR antibody (OX26)	PEG-BDNF-OX26	Restoration of CA1 region in hippocampus after ischemia in rats *in vivo*; i.v.	Wu and Pardridge, 1999
Targeting of transferrin receptor	Transferrin	PEG-Liposomes with BDNF gene-transferrin	Increased immunoreactivity of BDNF in cerebral cortex of rats compared to the background; i.v.	Xing et al., 2016
Targeting of lactoferrin receptor	Lactoferrin	PEG-PAMAM-GDNF-lactoferrin	Dose-dependent improvement in locomotor activity and reduced loss of TH+ neurons in SN of 6-OHDA-treated rats; i.v.	Huang et al., 2009
Targeting of LRP-1 receptor	Angiopep (19 aa)	Dendrigraft poly-L-Lys-PEG-GDNF gene-angiopep	Improved locomotor activity and recovery of dopamine neurons in rotenone rat PD model	Huang et al., 2013
Conjugation of NTF to cell penetrating peptide (CPP)	TAT (11 aa)	TAT-GDNF	Increased number of viable neurons in the striatum after ischemia; i.v.	Kilic et al., 2003
Conjugation of NTF to CPP	TAT	TAT-GDNF	TAT-GDNF fusion protein reached dopamine neurons but did not increase the number on TH+ neurons in mouse MPTP model; i.v.	Dietz et al., 2006
Bypassing of BBB	None	PEG-PLE-BDNF NPs	Active BDNF was released upon interaction with its receptors, minimizing potential side effects. Increased level of BDNF-containing NPs accumulated in the brain (compared to native BDNF) in all studied brain regions, except for midbrain. Delivery route not specified.	Jiang et al., 2018
Bypassing of BBB; Conjugation of NTF to CPP	TAT	Chitosan-TAT-GDNF	Increased number of viable TH+ neurons, decreased number of Iba-1 cells in MPTP-treated mice; i.n.	Hernando et al., 2018
Transient microbubbles (MB)-induced BBB opening	Ultrasound-responsive MB inducing transient opening of the BBB	MB-Liposomes with GDNF gene MB-Liposomes with BDNF gene MB-Liposomes with BDNF and GDNF genes	Improvement of behavioral deficits and rescued dopamine neuron loss in MPTP mice in GDNF, BDNF and GDNF+BDNF groups; i.v.	Lin et al., 2016, 2020
Magnetic resonance–guided transient BBB opening	Ultrasound-responsive MB inducing transient opening of the BBB	MB-PEG- polyethylenimine-GDNF gene	Localized delivery of GDNF to striatum; 11-fold increase in striatal GDNF in 6-OHDA-treated rats. 2.2-fold increase in DA levels, 3.2-fold increase in dopamine cell number in the SNpc and 5-fold increase in TH+ fiber density in the striatum at week 12 in 6-OHDA-treated rats. i.v.	Mead et al., 2017

* i.n. – intranasal delivery, i.v. – intravenous delivery.

#### Alternative Strategies: Bypassing the BBB or BBB Opening

As the delivery of NTFs to the brain using NPs functionalized with the BBB-targeting ligands did not lead to major breakthroughs just yet, various alternative strategies were applied to bypass or transiently open the BBB. In the relatively modern non-invasive delivery method relying on transient opening of the BBB, the surface of NPs can be functionalized with, e.g., microbubbles (MBs) that oscillate during the application of ultrasound, leading to transient opening of the BBB. Recently, MBs-functionalized liposomes with GDNF or/and BDNF genes were successfully delivered through the BBB *in vivo* in mice after i.v. administration and subsequent ultrasound-assisted transient BBB opening. Both GDNF and BDNF provided a neuroprotective effect in a mouse MPTP model of PD, with improvements shown in behavioral deficits and rescued dopamine neurons (Lin et al., 2016, 2020).

An alternative non-invasive delivery method bypassing the BBB is intranasal delivery (Aly and Waszczak, 2015) that can be combined with the targeted delivery. For example in the study by Hernando et al. (2018), human immunodeficiency virus 1 (HIV-1)-derived cell-penetrating transactivator of transcription (TAT) peptide was conjugated to chitosan-lipid NPs bearing GDNF and was tested in a mouse MPTP model of PD. The TAT-NP-GDNF-treated group revealed motor recovery, which was confirmed with immunohistochemistry studies showing increased number of dopamine neurons in the striatum as revealed by staining of tyrosine hydroxylase (TH), a marker of dopamine fibers and neurons.

An interesting intranasal delivery system was recently proposed by Jiang et al. (2018). The authors synthesized self-assembling PEG-PLE-BDNF NPs, in which active BDNF was released upon interaction with its receptors, minimizing potential side effects of BDNF caused by non-specific release. The authors administered NPs intranasally, and after 30 min detected an increased level of nano-BDNF accumulation in the brain (compared to native BDNF) in all studied brain regions, except for the midbrain, where was a trend for the increased native BDNF. The highest increase in BDNF levels was observed in the olfactory bulb (∼6.7 times), hippocampus (∼9.9 times), and brainstem (∼4.0 times) over native BDNF. Notably, in contrast to free BDNF, PEG-PLE-BDNF NPs significantly reduced dopamine neuron loss in the ipsilateral SN induced by intrastriatal injection of lipopolysaccharides.

One of the latest non-invasive delivery methods bypassing the BBB is a newly discovered methodology utilizing the brain lymphatic vasculature. Recent proof-of-concept study demonstrated that this method, which relies on subcutaneous injection of NPs at the neck near the lymph node, is 44-fold more efficient compared to conventional i.v. route for fluorescent-dye loaded PLGA NPs in mice (Zhao et al., 2020). To the best of our knowledge, this method has not yet been used for the delivery of NTFs through the BBB.

### Summary: The Potential of Nanoparticles for Improved NTF Delivery to the Brain

The NP-mediated delivery of NTFs to the brain is a complex phenomenon and its efficiency is a sum of several components: (a) stability of NTFs in the body fluids, (b) ability of NTFs to penetrate the BBB, and (c) diffusion of NTFs in the brain and their bioavailability to the target cells. Very few studies have so far addressed all of these components and mostly have reported the final result: improved locomotor behavior, reduced loss of TH+ neurons in the SN or the fold increase of NTF-NP concentration in the brain compared to the free NTFs. Below we elaborate on different components of successful NP-mediated delivery of NTFs to the brain and briefly summarize what may be achieved by the means of NPs.

Clearly, NPs can improve the stability of NTFs in the body fluids. While free NTFs are easily removed from circulation due to their rapid degradation by extracellular peptidases, tissue binding, receptor-mediated clearance, and glomerular filtration (Thorne and Frey, 2001), placement of NTFs into or onto NPs protects NTFs from enzymatic cleavage and tissue binding (physical protection) as well as glomerular filtration (glomerular filtration efficiently removes 1–30 kDa-sized peptides and proteins, while NPs increase their size). Conjugation of PEG to NTFs and NPs may further improve their pharmacokinetic properties and stability (Milton Harris and Chess, 2003).

Can NPs significantly improve the transportation of NTFs across the BBB? Although the types of NPs, time points, endpoints, animal models, and NP delivery methods are different in various studies and therefore cannot be compared in a straightforward manner, we have extracted relevant numbers from different articles to derive a sketch about the efficiency of current targeted systemic deliveries. Modest increases in NTF delivery by NPs were reported using large targeting ligands [transferrin: 2–3 times higher immunoreactivity of BDNF in cerebral cortex of rats compared to the background in naïve rats (Xing et al., 2016)] and surfactants (PX188): 1.5-fold higher radioactivity of ^125^I-CDNF-NP in the brain of naive rats compared to free ^125^I-CDNF (this study, Figure 6A). Promising results were reported by using the high ratio of short peptide angiopep conjugated to NPs: 3.06, 5.12 and 8.42-fold higher ^125^I-angiopep-NP radioactivity in the brains of mice compared to NPs without angiopep (Ke et al., 2009). The most promising results were reported for transient opening of the BBB, where an 11-fold increase in striatal GDNF protein was achieved in 6-OHDA-treated rats (Mead et al., 2017). Notably, the NP percentage in the brain compared to the delivered doses were still remarkably low in all identified studies and varied from 0.01 to 0.077%, whereas most of the NPs were localized in the spleen or liver (Sonavane et al., 2008; Prades et al., 2012; Rich et al., 2020; this study, Figure 6B). Thus, the efficiency of NPs in the transportation of NTFs across the BBB is still far from optimal.

Can nanoparticles facilitate spreading of NTFs inside the brain? The diffusion of free NTFs in the brain depends on the NTF size, charge, and presence of NTF receptors. The diffusion of smaller (<10 kDa) NTFs is not significantly restricted, while larger NTFs (>40 kDa) whose sizes begin to resemble the dimensions of extracellular space (15–40 nm), may encounter diffusion restrictions (Nicholson and Tao, 1993; Thorne and Frey, 2001). In addition, the presence of high-affinity receptors at NTF delivery sites may hinder the diffusion of NTFs. For example, Yan et al. (1994) injected radioactively labeled NGF, BDNF, and neurotrophin-3 (NT-3) into rat lateral cerebral ventricle and followed their distribution in the brain. While NGF reached the target cells, injection of BDNF resulted in few or no labeled neurons in the basal forebrain or in the SN. The authors concluded that the abundant expression of the BDNF receptor, *TrkB*, on the ependymal layer of the ventricle and brain parenchyma was the main reason behind the poor access of BDNF to its cellular targets.

Nanoparticles may help to overcome these difficulties. While NTFs diffuse in the extracellular space, NPs may follow the speedy cytoplasmic route through brain tissue. Since it is known that astrocytes make cytoplasmic bridges between different brain cells and connect a wide range of brain regions, it was suggested that astrocytes offer a “network of highways” that can be used by polymeric NPs to move inside the brain cells (Kreuter, 2014). PEGylation of NPs seems to facilitate the spreading of NPs inside the brain. It has been demonstrated *in vivo* and *ex vivo* in brain tissues that PEG-functionalized PLGA NPs diffuse in normal rat brain and PLGA NPs without PEG functionalization do not, whereas the percent of diffusive fractions correlated with the PEG surface density and NP size (Nance et al., 2012). Thus, PEGylated NPs might help to facilitate the diffusion of NTFs inside the brain that is especially useful for the NTFs such as GDNF that are readily bound by the components of extracellular matrix.

Nanoparticles can also mitigate other possible risks associated with the systemic delivery of NTFs, for example, possible side effects such as immune response or activation of receptors in “wrong” tissues. An intracerebroventricularly delivered GDNF trial was halted due to side effects (Nutt et al., 2003), which were likely due to extremely high GDNF doses affecting hypothalamus. Considering that GDNF receptors are expressed in different organs throughout the body, Manfredsson et al. (2020) suggested that GDNF administration for PD likely requires site-specific putaminal delivery that seems to be more advantageous over systemic delivery. However, targeted delivery with NPs would allow systemic treatment with NTFs by increasing the doses of NTFs in the brain and decreasing it, in the periphery. Since in PD dopamine neurons degenerate not only in the SN, but also in other brain regions, as well as in the periphery, it would be beneficial for NTFs to reach these neurons. In addition, in contrast to GDNF, CDNF, and MANF act mostly on stressed and diseased neurons and, therefore, their presence in the periphery could be beneficial for, e.g., enteric neurons (Lindahl et al., 2020) to relief the non-motor symptoms of PD such as constipation.

Despite initial encouraging results on safety of some NPs and their approval by FDA and EMA, potential side effects of NPs in the context of neurodegenerative diseases should be thoroughly explored. As stated in the recent review, no single clinical trial based on the use of NPs to treat PD has been registered yet (Torres-Ortega et al., 2019). Authors concluded that the scarcity of validated methods for characterization of NPs, manufacturing issues and safety and clinical translation concerns are main current limitations of nanotechnologies for PD therapy that should be considered during development of nanomedicines for PD.

## Perspective

### Nanoparticle-Mediated Drug Delivery for Emerging Approaches

While NTFs maintain and restore dopamine neurons that are damaged but not lost, cell transplants are suggested to restore the lost cells. Relatively large number of clinical transplantation trials using developing dopamine neurons from human fetal midbrain has been conducted. Although the outcomes were positive in some PD patients, there were numerous problems in other patients such as graft-induced dyskinesia or propagation of pathological αSyn aggregates into the grafted cells (Li et al., 2008). Recently, the first studies using human stem cell-derived and pluripotent stem cell-derived dopamine neurons were conducted (Barker et al., 2018; Schweitzer et al., 2020; Tao et al., 2021). A key requirement for cell-replacement therapy to be successful is that engrafted neurons stay alive and integrate into resident neuronal networks by forming new synaptic contacts (Parmar et al., 2020). NTFs delivered systemically with NPs could serve as a supporting tool in these therapies, keeping neurons alive and facilitating the establishment of neuronal contacts (Gantner et al., 2020).

Another PD therapy is the use of small molecules that mimic the action of NTFs, NTF mimetics, e.g., GDNF family ligands (GFLs) that promote the survival of different neuronal populations. Despite their promising neurorestorative properties, these molecules still suffer from poor pharmacological characteristics, do not efficiently penetrate the BBB, and activate several receptors in different cell types (Sidorova and Saarma, 2020). NPs could improve the physico-chemical properties (such as solubility) and BBB penetration properties of these molecules.

A modern potential treatment for PD is reprogramming of brain cells, such as *in vivo* conversion of astrocytes into dopamine neurons that currently relies on genes of transcription factors that are delivered into the brain using lentiviruses injected into the striatum (Rivetti Di Val Cervo et al., 2017). Similarly, reprogramming of glial cells into neurons using CRISPR-CasRx has recently been demonstrated using Ptbp1 knockdown in the striatum (Zhou et al., 2020). These modern techniques rely on the delivery of DNA and RNA that could be delivered systemically using NPs (Wei et al., 2020).

### Dual Functionality of NPs

In addition to their nanocarrier function, NPs themselves have been increasingly shown to support various functions of neurons, prevent the aggregation of proteins, reduce inflammation, and alleviate endoplasmic reticulum (ER) stress caused by pathological processes in the brain of patients with neurodegenerative diseases. This emerging dual strategy that combines drug carrier and therapeutic function of NPs may be of significance.

#### Nanoparticles as Neuroprotective and Anti-Inflammatory Agents

Several research articles reported improved growth, differentiation or survival of neuronal cells and neurons on nanomaterials, especially on carbon-based materials such as graphene (Convertino et al., 2020), carbon nanotubes (Shao et al., 2018), and nanodiamonds (Alawdi et al., 2017). Some forms of graphene oxide prevented the loss of dopamine neurons and decreased αSyn levels *in vitro* in the cell line SN4741 derived from the murine SN (Rodriguez-Losada et al., 2020). Graphene promoted axonal elongation by reducing the number of retrogradely transported NGF in the culture of primary dorsal root ganglion neurons (Convertino et al., 2020). Nanodiamonds decreased inflammation, improved learning, and stimulated expression of BDNF in an aluminum-induced rat model of AD, probably *via* modulation of the NF-κB pathway (Alawdi et al., 2017). Similarly, several inorganic NPs were neuroprotective against inflammation and reactive oxygen species (ROS). For example, selenium NPs reversed oxidative damage and neuronal loss in a mouse model of epilepsy (Yuan et al., 2020) and cerium oxide NPs blocked pro-inflammatory signaling and drove microglial transformation from a pro-inflammatory phenotype to an anti-inflammatory phenotype under pathological conditions in microglial cell line BV-2 (Zeng et al., 2018). Similarly, biomimetic NPs coated with the cell membranes of neuronal cells promoted the transformation of microglia into the anti-inflammatory phenotype to relieve neuroinflammation and recover dopamine levels in a mouse model of PD (Liu et al., 2020). Thus, some NPs could serve as therapeutics by reducing ROS and inflammation which underlie neurodegenerative diseases.

#### Nanoparticles as Inhibitors of Protein Aggregation

Aggregation of proteins into pathological fibrils and subsequent ER stress caused by misfolded proteins are common hallmarks of a range of neurodegenerative diseases including Aβ in AD and αSyn in PD. Inhibiting protein amyloid aggregation has become one of the popular approaches to potentially treat AD and PD. Several articles have recently demonstrated that Au and carbon-based NPs efficiently penetrated the BBB and, in addition to their nanocarrier function, prevented and reversed αSyn aggregation in PD models and Aβ aggregation in AD models. It has been demonstrated that graphene quantum dots not only prevented formation of αSyn fibrils but also disassembled formed fibrils (Kim et al., 2018). Specifically, the authors of the latter study demonstrated that the protective effects of quantum dots were also valid *in vivo* in an αSyn fibrils-induced mouse model of PD: stereotaxically injected αSyn provoked accumulation of fibrils in the striatum and SN but systemic administration of graphene quantum dots reduced the levels of phosphorylated αSyn in murine brain and reduced behavioral deficits. The mechanism of pathological fibrillar remodeling is not fully understood but it is most probably attributed to the reduction of β-sheets and increase of α-helices and random coils in the secondary structure of αSyn fibrils by graphene quantum dots. Since quantum dots contained a high number of COOH groups on their surface, these groups could interact with the positively charged N-terminal domain of α-Syn and facilitate disaggregation of αSyn fibrils (Kim et al., 2018). Similar findings have been reported for Aβ-forming fibrils and plaques in AD, where Au NPs, graphene and carbon dots inhibited Aβ fibrillation and remodeled previously formed fibrils (Mahmoudi et al., 2012; Javed et al., 2019; Zhou et al., 2019), probably by clearance and extracting peptides from Aβ fibrils (Yang et al., 2015). Javed et al. (2019) demonstrated that Au NPs sequestered intracerebral Aβ due to their capacity to bind with misfolded proteins in a chaperone-like manner. All above-mentioned NPs bear a significant potential for the treatment of protein-aggregation-related neurodegenerative diseases *via* dual strategies combining the properties of NPs as drug carriers and as drugs themselves to mitigate protein aggregation and ER stress.

## Author Contributions

Both authors planned and wrote the manuscript, and approved the final version of the manuscript.

## Conflict of Interest

MS is the inventor in the MANF- and CDNF-related patents owned by Herantis Pharma Plc. OB is the shareholder in Herantis Pharma Plc.
